# Humic Substances Alter Ammonia Production and the Microbial Populations Within a RUSITEC Fed a Mixed Hay – Concentrate Diet

**DOI:** 10.3389/fmicb.2018.01410

**Published:** 2018-07-02

**Authors:** Stephanie A. Terry, Aline F. O. Ramos, Devin B. Holman, Tim A. McAllister, Gerhard Breves, Alexandre V. Chaves

**Affiliations:** ^1^School of Life and Environmental Sciences, Faculty of Science, The University of Sydney, Sydney, NSW, Australia; ^2^Animal Science Graduate Course, Veterinary Medicine Institute, Federal University of Pará, Belém, Brazil; ^3^Lacombe Research and Development Centre, Agriculture and Agri-Food Canada, Lacombe, AB, Canada; ^4^Lethbridge Research and Development Centre, Agriculture and Agri-Food Canada, Lethbridge, AB, Canada; ^5^Department of Physiology, University of Veterinary Medicine Hannover, Hanover, Germany

**Keywords:** humic substances, methane, cattle, microbiome, ruminant nutrition, rumen stimulation technique

## Abstract

Humic substances are a novel feed additive which may have the potential to mitigate enteric methane (CH_4_) production from ruminants as well as enhance microbial activity in the rumen. The aim of this study was to examine the effects of humic substances on fermentation characteristics and microbial communities using the rumen stimulation technique (RUSITEC). The experiment was conducted as a completely randomized design with 3 treatments duplicated in 2 runs (a 15-day period each run) with 2 replicates per run. Treatments consisted of a control diet (forage:concentrate; 60:40) without humic substances or humic substances added at either 1.5 g/d or 3.0 g/d. Dry matter disappearance, pH, fermentation parameters and gas production were measured from day 8 to 15. Samples for microbial profiling were taken on day 5, 10, and 15 using the digested feed bags for solid- associated microbes (SAM) and fermenter fluid for liquid- associated microbes (LAM). The inclusion of humic substances had no effect (*P* ≥ 0.19) on DM disappearance, pH or the concentrations of VFA. The production of NH_3_ was linearly decreased (*P* = 0.04) with increasing levels of humic substances in the diet. There was no effect (*P* ≥ 0.43) of humic substances on total gas, CO_2_ or CH_4_ production. The number of OTUs was significantly reduced in the 3.0 g/d treatment compared to the control on d 10 and 15; however, the microbial community structure was largely unaffected (*P* > 0.05). In the SAM samples, the genera *Lachnospiraceae* XPB1014 group, *Succiniclasticum*, and *Fibrobacter* were reduced in the 3.0 g/d treatment and *Anaeroplasma, Olsenella*, and *Pseudobutyrivibrio* were increased on day 5, 10, and 15. Within the LAM samples, *Christensenellaceae* R-7 and *Succiniclasticum* were the most differentially abundant genera between the control and 3.0 g/d HS treatment samples (*P* < 0.05). This study highlights the potential use of humic substances as a natural feed additive which may play a role in nitrogen metabolism without negatively affecting the ruminal microbiota.

## Introduction

Livestock are known to be large contributors to global greenhouse gas emissions. A recent report by Wolf et al. (2017) proposed that emissions from livestock have been underestimated by 11% when using criteria reported by IPCC (2006) including an underestimation of 8.4% in predicted enteric CH_4_ fermentation (Wolf et al., 2017). Not only is CH_4_ a potent greenhouse gas, it also represents a 2–12% loss of gross energy consumed (Johnson and Johnson, 1995). There are many strategies for reducing CH_4_ emissions from ruminants, with the use of feed additives still regarded as one of the most promising methods of mitigation in intensive production systems.

Humic substances (HS) are geological deposits made of a mixture of complex acids which arise from the natural decomposition of animal and plant material (McMurphy et al., 2009). Humic and fulvic acids are the major extractable components of soil humates and are most commonly used to improve soil fertility (Rajendiran and Purakayastha, 2016). Humic substances have been shown to have antimicrobial activity (Váradyová et al., 2009; Degirmencioglu, 2012) as well as absorptive and detoxifying properties (Islam et al., 2005). In soils, HS promote microbial growth (Huck et al., 1991) and it has been proposed that they may have a similar effect within the rumen, enhancing microbial activity and increasing fermentation.

Humic substances have been shown to act as electron acceptors for a large variety of microorganisms capable of extracellular electron transfer, including methanogens (Martinez et al., 2013). Humic substances contain functional structures including quinone and phenolic hydroxyl as well as molecules containing nitrogen and sulfur which are involved in its redox function (Aeschbacher et al., 2010). Evidence of the CH_4_ reducing effect of these functional structures within the rumen were shown by Sheng et al. (2017) who examined HS in an *in vitro* batch culture and found that HS consistently decreased CH_4_ production when included at up to 3.6 mg/mL of inoculum during a 48 h incubation.

Existing studies have been inconclusive when evaluating the effect of HS compounds in ruminant diets (Váradyová et al., 2009; McMurphy et al., 2011; Degirmencioglu, 2012). This variability may be attributed to variation in the chemical properties of HS among sources, extraction methodologies, dosage, and concentrations of other vitamins and minerals in the diet (Islam et al., 2005). However, this study will examine the same source of HS as used by Sheng et al. (2017) who demonstrated that dry matter disappearance and microbial synthesis were increased and NH_3_-N production was decreased by HS in *in vitro* batch cultures.

In the present study, we hypothesized that inclusion of HS would decrease CH_4_ emissions and alter the ruminal microbial community. As such, the objective of this study was to examine the effect of two concentrations of HS on fermentation characteristics, CH_4_ production and microbial populations using the rumen stimulation technique.

## Materials and Methods

The donor cows used in this experiment were cared for in accordance with the guidelines of the German Animal Welfare Act approved by the Lower Saxony State Office for Consumer Protection and Food Safety (LAVES, approval number AZ 33.4-42505-04-13A373).

### Experiment Design and Treatments

The experiment was conducted as a completely randomized design with 3 treatments duplicated in 2 runs with 2 replicates per run. The 3 treatments consisted of a control diet (no HS inclusion) and two different inclusions of HS in the diet (DM basis) fed at 1.5 and 3.0 g/d. The HS were obtained from Canadian Humalite International Inc. and contained 50.7% humic acids and 4.4% fulvic acids. Dietary concentrations of HS were selected based on a preliminary batch fermentation study (Sheng et al., 2017) which found that CH_4_ production was decreased and DM disappearance was increased when HS were included at up to 3.6 mg/mL of inoculum culture. The experimental period consisted of 15 days with day 1–7 used for adaptation and day 8–15 used for measurements.

The substrate used was a hay:concentrate (60:40 DM basis) diet using hay obtained from natural grassland of Lower Saxony, Germany. Hay was prepared using an electrical clipper with a 76- mm blade (Duarte et al., 2017). The commercial concentrate was pelleted (Deuka Schaffutter, Deutsche Tiernahrung Cremer, Düsseldorf, Germany) and contained 20% crude protein, 2.6% crude fat, 11.0% crude fiber, 8.7% crude ash, 0.9% calcium, 0.55% phosphor and 0.2% sodium. Both the hay and substrate were weighed into the same nylon bag (10 cm × 5 cm, pore size 50 ± 10 μm) for a total mass of 11 g of substrate. The HS were placed in a separate nylon bag (5.0 cm × 2.5 cm, pore size 150 μm) to the substrate.

### Inoculum Sampling and Incubation Procedure

Rumen inoculum was obtained from two ruminally cannulated Holstein heifers, 2 h after morning feeding. Cattle were fed hay (e.g., same hay used as substrate) *ad libitum* and 600 g/d of a commercial concentrate (Deuka Schaffutter, Deutsche Tiernahrung Cremer, Düsseldorf, Germany). Rumen contents were separated into rumen fluid and solid rumen contents by gauze filtration. Samples for DNA extraction were collected from the solid (15 g) and liquid proportions (40 mL) from each cow and immediately frozen in liquid nitrogen. Samples were stored at −40°C until extraction.

Fluid samples from each heifer were pooled together and the pH and redox potential was recorded. Samples (2 mL) were also taken and stored at −20°C for determination of volatile fatty acids (VFA) and ammonia (NH_3_).

The incubation procedure was conducted as described by Czerkawski and Breckenridge (1977). Prewarmed 800 mL fermentation vessels were placed in the Rusitec apparatus and water was kept at 39°C. Each fermentation vessel had an inner vessel which contained one nylon bag containing 70 g of solid digesta, one bag with the basal diet, and one small bag containing the HS. Each fermenter was filled with approximately 750 mL of rumen fluid and infused with McDougall’s buffer at a dilution rate 30 mL/h. The inner vessels were continuously moved up and down by an electric motor to ensure adequate mixing between fluid and particles. After the first 24 h of incubation, the bag with the solid rumen digesta was replaced with a bag containing the diet. Bags were replaced with a fresh bag containing feed after 48 h of incubation, replacing 1 bag per day. Bags from day 15 were not used for DM determination as they were only incubated for 24 h. Effluent was collected in 2 L glass flasks which were kept on ice to arrest microbial growth and impede fermentation.

### Sample Collection

Dry matter disappearance (DMD) at 48 h was determined on day 8 and 10–13 when bags were not used for DNA extraction. After removal from the vessel, feed bags were washed in 50 mL of warmed buffer in a small plastic bag, gently squeezed and the residual buffer was placed back into the fermenter to ensure transfer of solid-phase-associated microorganisms. The residual feed bag was rinsed under cold water until the water was clear and then dried at 55°C for 48 h for the determination of DMD (Duarte et al., 2017). After drying, substrate samples from day 9, 10, and 14 were taken from the bag and ground using a coffee grinder for NDF analysis.

Total daily gas production was collected in gas-tight bags (Plastigas, Linde AG, Munchen, Germany). From day 8 to 15, before measurement of total gas, two 20 mL aliquots were taken from the septum of each gas bag and transferred into evacuated tubes for the analysis of CH_4_ and CO_2_. Total daily gas production was measured using a drum-type meter (Ritter Apparatebau, Bochum, Germany).

During bag exchange, fermenter pH, gas production and effluent volume for each fermenter was measured. The pH and redox potential of the vessel was measured daily during bag exchange using a Knick pH meter (digital pH meter 646, Knick, Berlin, Germany). Effluent from each fermenter was measured and two samples (2 mL) of effluent were taken and stored at −40°C until analyzed for VFA and NH_3_.

### DNA Extraction and Sequencing of the 16S rRNA Gene

On day 5, 10, and 15 nylon bags, as well as 30 mL of fermenter liquid were removed from each vessel and immediately placed in liquid nitrogen for later extraction of DNA. Samples were stored at −40°C until they were placed in a freeze dryer (48 h solid samples, 72 h liquid samples). Samples were then finely ground using a coffee grinder and placed back into the freezer until extraction. The liquid samples were freeze dried for 4 days and then ground using a mortar and pestle.

Total DNA was extracted from each sample using a QIAamp Fast DNA stool mini kit (Qiagen, Hilden, Germany), according to the manufacturer’s instructions. DNA yield and purity was measured using a NanoDrop spectrophotometer (Thermo Fisher Scientific, Waltham, MA, United States). Extracted DNA was stored at −20°C until sequencing.

The V4 hypervariable region of the archaeal and bacterial 16S rRNA gene was amplified using the modified 515-F and 806-R primers as described by Walters et al. (2016). The PCR conditions and sequencing steps were as previously detailed (Duarte et al., 2017). Briefly, the 16S rRNA gene amplicons were generated using a two-step PCR and sequenced on an Illumina MiSeq instrument (Illumina, Inc., San Diego, CA, United States) using the MiSeq Reagent Kit v2 (500 cycles; Illumina, Inc.), and according to manufacturer’s instructions.

The R-package DADA2 (v. 1.4) was used to process the 16S rRNA gene sequences. This included primer removal and truncating both the forward and reverse reads at 225 bp. Based on quality scores, the number of expected errors allowed per read was of 2 and no ambiguous base calls were permitted. Reads were then merged and chimera sequences removed. The RDP naïve Bayesian classifier (Wang et al., 2007) and the SILVA SSU database v. 128 (Quast et al., 2012) with a 50% bootstrap confidence threshold were used to assign taxonomy to each inferred 16S rRNA gene sequence; that is, an operational taxonomic unit (OTU) at 100% similarity. Richness (number of OTUs) and diversity (Shannon index) were calculated using QIIME v. 1.9.1 (Caporaso et al., 2010). The R packages vegan (v. 2.4.4; Oksanen et al., 2017) and phyloseq (v. 1.20.0; McMurdie and Holmes, 2013) were used to calculate and plot principal coordinates analysis (PCoA) of Bray–Curtis dissimilarities.

### Chemical Composition

Feed was analyzed, following AOAC (2005) methods, for DM (method 967.03). Neutral detergent fiber (NDF) content was analyzed according to Van Soest et al. (1991) with the use of sodium sulfite and heat-stable α-amylase. Methane and CO_2_ was measured by using gas chromatography (GC 2014, Shimadzu Europa GmbH, Duisburg, Germany) and CH_4_ and CO_2_ production was calculated by multiplying the total gas volume by the percentage of CH_4_ with correction for temperature and pressure (0°C’, 101.3 kPa; Riede et al., 2013).

All 16S rRNA gene sequences were deposited into the NCBI Sequence Read Archive under BioProject accession PRJNA436853 (SAMN08634222 to SAMN08634277).

### Statistical Analysis

The univariate procedure in SAS was used to test for normal distribution of data. Data was analyzed as a completely randomized design using the PROC MIXED procedure of SAS (SAS Institute, 2018). Day and treatment were considered fixed effects, with day used as a repeated measure. Data from replicate vessels were averaged prior to statistical analysis and these averages, within run, were considered the statistical unit. The minimum values of Akaike’s information criterion were used to select the covariance structure. Linear and quadratic effects were evaluated by using planned orthogonal polynomial coefficients for each parameter when Type 3 tests for fixed effects were ≤0.05. Significance among treatments was declared at *P* ≤ 0.05.

Prior to analysis, all samples were randomly subsampled to 16,000 sequences to account for differences in sequencing depth. The archaeal and bacterial community structure was analyzed using permutational multivariate analysis of variance (PERMANOVA) and the adonis function with 10,000 permutations in the R package vegan (v. 2.4.4; Oksanen et al., 2017; R Core Team, 2017). The betadisper function in vegan was used to assess the homogeneity of dispersion for each time point. Linear discriminant analysis effect size (LEfSe; Segata et al., 2011) was used to identify genera with a relative abundance of greater than 0.1% that were differentially abundant between the control and humic acid 3.0 g/d treatments for both SAM samples at day 5, 10, and 15, and LAM samples for day 10 and 15. A minimum LDA score of 3.5 was used as the threshold for classifying differentially abundant genera.

## Results

### Effect of Humic Substances on *in Vitro* Fermentation

The chemical compositions of the diet and HS are shown in **Table 1**. The addition of HS had no effect (*P* ≥ 0.19) on DM disappearance, pH, redox or VFA production (**Table 2**). Ammonia production was linearly decreased (*P* = 0.04) with the addition of HS to the diet. HS had no effect (*P* ≥ 0.43) on total gas production, CO_2_ or CH_4_ production (**Table 3**).

**Table 1:** Chemical composition of hay, concentrate and humic substance (%DM).

	Hay	Concentrate^1^	Humic substances^2^
Dry matter (DM)	92.1	92.7	75.7
Neutral detergent fiber (NDF)	69.7	44.1	
Ash	5.53	4.54	24.6

*^1^The commercial concentrate was produced by Deuka Schaffutter, Deutsche Tiernahrung Cremer, Düsseldorf, Germany, 20% crude protein, 2.6% crude fat, 8.7% crude ash, 0.9% calcium, 0.55% phosphor and 0.2% sodium. ^1^Humic Substance was manufactured by Canadian Humalite International Inc. Edmonton, Canada; 8.64 g/kg Ca, 1.23 g/kg Mg, 2.13 g/kg Fe, 0.34 g/kg K, 1.41 g/kg Na, 51.3 g/kg S, 0.14 g/kg Mn, 0.34 g/kg K, 0.31 g/kg Ti, 5270 mg/kg Al, 1.47 mg/kg As, 0.165 mg/kg Cd, and 11.6 mg/kg Pb.*

**Table 2:** Effect of humic substance on dry matter (DM) disappearance, pH, redox, quantity of individual volatile fatty acids (VFA) and ammonia produced over a 24 h period in a Rusitec fed a mixed hay – concentrate diet.

	Concentration HS (g/d)				*P*-value		
	Control	1.5	3.0	SEM	Treatment	Linear	Quadratic
DM disappearance (%)	47.1	48.2	48.9	0.75	0.19	0.07	0.86
pH	6.80	6.80	6.79	0.013	0.77	0.50	0.81
Redox	255.9	260.7	255.6	6.73	0.84	0.97	0.56
Acetate (A, mmol/day)	10.5	10.4	10.6	0.46	0.97	0.87	0.87
Propionate (P, mmol/day)	5.04	4.84	5.07	0.192	0.67	0.91	0.38
Butyrate (mmol/day)	2.53	2.47	2.50	0.204	0.98	0.92	0.86
Valerate (mmol/day)	0.65	0.69	0.54	0.065	0.27	0.23	0.28
A:P ratio	2.09	2.17	2.11	0.086	0.81	0.89	0.54
NH_3_-N (mmol/day)	4.82^b^	4.41^a^	4.53^a^	0.095	0.02	0.04	0.04
Daily effluent volume (L/day)	0.70	0.66	0.70	0.021	0.41	0.95	0.20

*HS, humic substances. Different lowercase letters in rows indicate significantly different means (P < 0.05). Daily production of individual VFA or ammonia = daily effluent volume (L/day) × concentration (mmol/L).*

**Table 3:** Effect of humic substance on gasses production in a Rusitec fed a mixed hay – concentrate diet.

	Concentration HS (g/d)				*P*-value		
	Control	1.5	3.0	SEM	Treatment	Linear	Quadratic
Total gas (mL/d)	800.0	840.0	900.0	53.01	0.43	0.21	0.83
CO_2_ (mL/d)	66.7	69.7	79.1	7.76	0.51	0.28	0.74
CO_2_ (mg/d)	131.0	136.9	155.5	15.26	0.51	0.28	0.74
CO_2_ (mg/g DM disappeared)	21.5	24.1	26.8	4.59	0.73	0.44	0.99
CH_4_ (mL/d)	23.6	22.8	26.1	2.97	0.72	0.57	0.58
CH_4_ (mg/d)	16.9	16.3	18.6	2.15	0.72	0.57	0.58
CH_4_ (mg/g DM disappeared)	2.89	2.90	3.26	0.623	0.89	0.68	0.82

*HS, humic substances.*

### Effect of Humic Substances on the Rumen Microbiota

A total of 4,512,841 16S rRNA gene sequences were obtained which were classified into 128 families (96.5% of sequences) and 296 genera (82.8% of sequences). The microbial community structure of the LAM and SAM differed (*R*^2^ = 0.08; *P* < 0.0001). *Prevotella*, *Megasphaera*, *Rikenellaceae* RC9 gut group, *Fibrobacter*, *Lactobacillus*, and *Treponema* were among the 10 most abundant genera in both SAM and LAM Rusitec contents (Supplementary Figures S1, S2).

**FIGURE 1:**
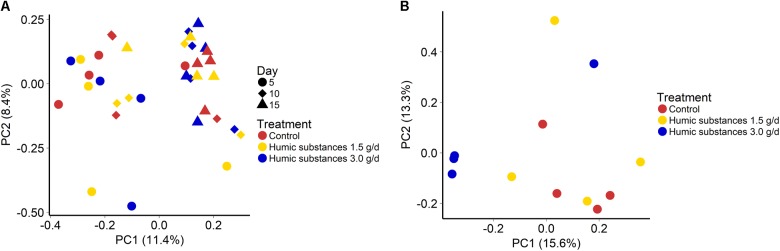
Principal coordinates analysis (PCoA) plots of the Bray–Curtis dissimilarities for **(A)** solid-associated microbe (SAM) samples by treatment (PERMANOVA: *P* = 0.43) and sampling time (*R*^2^ = 0.11; *P* < 0.0001) and **(B)** liquid-associated microbe (LAM) samples by treatment (*P* = 0.12) at day 15. Percentages of variation explained by the principal coordinates are indicated on the axes.

The addition of HS at 1.5 or 3.0 g/d did not alter the community structure in either the SAM (**Figure 1A**; *P* = 0.43) or LAM (**Figure 1B**; *P* = 0.12) samples. However, sampling time exhibited an effect on the microbiota within the SAM samples (**Figure 1A**; *R*^2^ = 0.11; *P* < 0.0001). Overall, the three treatments were more similar on day 15 than on day 5 and 10 (Supplementary Figure S3). Although there was no clustering by treatment group within the LAM samples (day 15), HS at 3.0 g/d did alter the microbial community structure when compared with the control and 1.5 g/d treatment (Supplementary Figure S4; *P* < 0.05).

**FIGURE 2:**
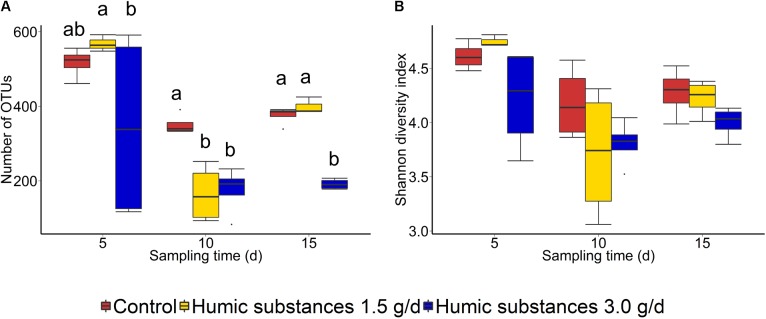
Box plots of the **(A)** number of OTUs and **(B)** Shannon diversity index for solid-associated microbe (SAM) samples by treatment and sampling time. Different lowercase letters within each sampling time indicate significantly different means (*P* < 0.05).

**FIGURE 3:**
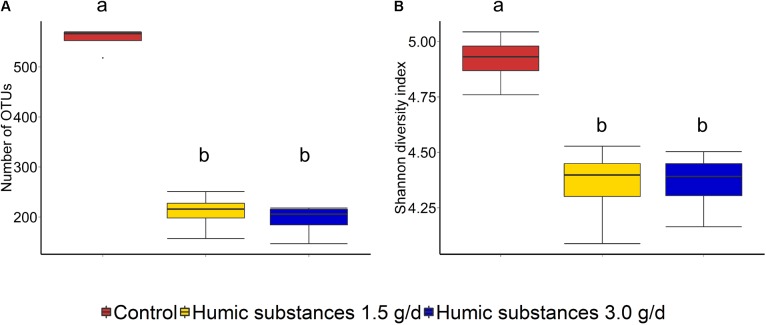
Box plots of the **(A)** number of OTUs and **(B)** Shannon diversity index for liquid-associated microbe (LAM) samples by treatment at day 15. Different lowercase letters within each sampling time indicate significantly different means (*P* < 0.05).

Within the SAM fraction, the number of OTUs was reduced in the 3.0 g/d HS treatment compared to the control on day 10 and 15 (**Figure 2A**), although microbial diversity (Shannon diversity index) was unaffected (**Figure 2B**; *P* > 0.05). Compared to the control samples, both the number of OTUs and the Shannon diversity index were decreased (*P* < 0.05) by HS in the LAM samples taken on day 15 (**Figure 3**).

Differentially abundant genera were identified between the control and the 3.0 g/d HS treatment for both SAM and LAM samples (**Table 4**). Within the LAM samples, *Christensenellaceae* R-7 and *Succiniclasticum* were the most differentially abundant genera between the control and 3.0 g/d HS treatment samples (*P* < 0.05).

**Table 4:** Differentially abundant genera identified between the control and 3.0 g humic substances per day for solid associated microbes (SAM) on day 5, 10 and 15 and liquid associated microbes (LAM) on day 15 in a Rusitec fed a mixed hay – concentrate diet.

	Relative abundance (%)		
Genus	Control	3.0 g HS/d	LDA score
SAM d 5			
*Lachnospiraceae* XPB1014 group	**0.58 ± 0.04**	0.16 ± 0.07	3.8
*Succiniclasticum*	**1.59 ± 0.15**	0.88 ± 0.28	3.7
SAM d 10			
*Escherichia*/*Shigella*	0.02 ± 0.01	**1.20 ± 0.49**	3.8
*Anaeroplasma*	0.17 ± 0.10	**0.90 ± 0.10**	3.7
*Veillonellaceae* UCG 001	**1.11 ± 0.04**	0.73 ± 0.18	3.6
*Ruminococcaceae* UCG 002	**0.32 ± 0.10**	0.08 ± 0.02	3.5
SAM d 15			
*Fibrobacter*	**11.49 ± 1.31**	4.89 ± 0.28	4.6
*Ruminococcaceae* UCG 010	**0.16 ± 0.02**	0.04 ± 0.02	3.7
*Olsenella*	0.18 ± 0.03	**0.80 ± 0.25**	3.7
*Pseudobutyrivibrio*	0.99 ± 0.07	**1.34 ± 0.12**	3.7
*Ruminococcaceae* NK4A214 group	**0.35 ± 0.07**	0.17 ± 0.05	3.5
*Papillibacter*	**0.22 ± 0.05**	0.09 ± 0.01	3.5
LAM d 15			
*Christensenellaceae* R7 group	**1.74 ± 0.23**	0.56 ± 0.13	3.9
*Succiniclasticum*	**1.71 ± 0.10**	0.81 ± 0.32	3.8
*Methanomicrobium*	0.005 ± 0.005	**0.84 ± 0.43**	3.8
*Prevotellaceae UCG 003*	**2.83 ± 0.30**	1.80 ± 0.26	3.8
*Schwartzia*	0.46 ± 0.07	**1.19 ± 0.33**	3.8
*Coprococcus*	**0.22 ± 0.03**	0.02 ± 0.01	3.7

*HS, humic substances; LDA = linear discriminant analysis. Bold text indicates higher abundance of genera. Values represent the percent relative abundance of each genus.*

Notably, *Methanomicrobium*, a genus of methane-producing archaea, was enriched in the 3.0 g/d HS treatment. However, the relative abundance of the archaeal class *Methanobacteria* did not differ by treatment or sampling time (*P* > 0.05). In the SAM samples, *Lachnospiraceae* XPB1014 group (day 5), *Succiniclasticum* (day 5), and *Fibrobacter* (day 15), were among those genera with higher abundance in the control while *Anaeroplasma* (day 10), *Escherichia* (day 10), *Olsenella* (day 15), and *Pseudobutyrivibrio* (day 15) were depleted compared to the 3.0 g/d of HS.

## Discussion

The HS used in this experiment contained 50.7 and 4.4% of humic and fulvic acids, respectively. Other studies examining the use of HS as a feed additive in ruminant diets have reported organic acid concentrations ranging from 61.8 to 89.8% (Cusack, 2008; Váradyová et al., 2009; McMurphy et al., 2011). Comparatively, the concentrations used are of low to mid-range. This presents a difficulty when discussing results of other studies as the source, preparation and organic content of HS may influence their effects on ruminal fermentation (Islam et al., 2005).

Disappearance of DM or VFA concentrations was not affected by HS. This is in contrast to results observed by Sheng et al. (2017) who found that HS increased DMD and decreased production of acetate when included at the 3.6 mg/mL of inoculum in a ruminal batch culture, the same concentration used in this study (Sheng et al., 2017). The inclusion of humic substances linearly decreased NH_3_ production by 8.5%, a finding that agrees with Sheng et al. (2017) who demonstrated that NH_3_-N concentration was decreased after 12 h incubation in *in vitro* batch cultures. Váradyová et al. (2009) found that when humic acids were added at 10 g/kg DM to a forage based diet, that NH_3_-N production was reduced by 24.4%. Ammonia is the product of protein degradation within the rumen and rumen bacteria utilize NH_3_-N as a nitrogen source for growth (Koenig et al., 2000; Bach et al., 2005). Reductions in NH_3_ concentration could reflect an improvement in the efficiency of microbial protein synthesis (Bach et al., 2005). Váradyová et al. (2009) reported that nitrogen incorporated by microbiota and the efficiency of microbial synthesis was increased by humic acid in a high concentrate diet (Váradyová et al., 2009). The decrease in NH_3_ production may be a result of the antimicrobial properties of HS on protozoa, which can engulf rumen bacteria which utilize NH_3_-N for their nitrogen requirements (Degirmencioglu, 2012).

There is evidence that HS reduce CH_4_ in soils and in the rumen using *in vitro* experimental techniques (Sheng et al., 2017; Tan et al., 2018). Tan et al. (2018) indicated that CH_4_ was suppressed by up to 40% in anoxic environments when humic acid was included in batch incubations at 60 mg/L of total solution with 40 g of wet soils. Sheng et al. (2017) found that CH_4_ was decreased by 12.8% over 48 h of incubation when HS was included at up to 3.6 mg/mL of incubation fluid from cattle. Humic substances are redox-active and have potential to act as terminal electron acceptors in anaerobic microbial respiration (Martinez et al., 2013). However, in the current study, there was no effect of HS on CH_4_ production. This result agrees with Váradyová et al. (2009) who found, using rumen fluid from a sheep, that CH_4_ was not changed as a result of inclusion of HS up to 20 g/kg DM and 10 g/kg DM using batch culture and Rusitec techniques, respectively (Váradyová et al., 2009).

This discrepancy in results may be attributed to differences in the organic content and chemical structures of the HS (Islam et al., 2005). However, Sheng et al. (2017) used the same HS product as our present study and differences in these results may demonstrate the advantages of the semi-continuous rumen stimulation technique over *in vitro* batch cultures (Hristov et al., 2012). Batch cultures are short experiments usually conducted over a 24–48 h period, allowing little adaptation of microbes to treatment. The RUSITEC, however, is conducted over a longer period allowing adaptation of microbes to the system, enabling closer representation of the rumen. This suggests that perhaps microbes were able to adapt to the presence of HS in the RUSITEC. It seems that while there is the chemical potential for HS to decrease CH_4_ emissions in an anaerobic environment, within the complex nature of the rumen, HS do not affect CH_4_ metabolism. This lack of change in CH_4_ production is further supported by the fact that the relative abundance of methanogens did not differ among treatments.

As expected based on previous work (de Menezes et al., 2011; Duarte et al., 2017) sample type (LAM vs. SAM) had a significant effect on the structure of the rumen microbiota. Incubation time had the strongest effect on the microbial community (**Figure 1A**) and samples did not cluster by HS treatment. Microbial richness (number of OTUs) was significantly reduced compared to the control in both HS treatments and the Shannon diversity index was also decreased in the LAM samples (**Figures 2**, **3**). This finding was due to the loss of rare taxa (<0.1%) that may have been more sensitive to HS rather than large changes in the abundance of the more prevalent taxa.

Predominant phyla present within the rumen include members of *Firmicutes* and *Bacteroidetes* (Seshadri et al., 2018). In contrast, genera of *Fibrobacter, Lactobacillus*, and *Megasphaera* dominated the microbiome within this RUSITEC. However, there have been reports of similar abundances in other RUSITEC experiments (Wetzels et al., 2018; Ramos et al., unpublished). This change in dominant phyla is likely the result of the artificial environment, where these are less susceptible to change in environment. Interestingly Mateos et al. (2017) found through quantitative PCR that *Fibrobacter succinogenes* were linearly decreased throughout 14 days within a RUSITEC, compared to the current experiment where this phylum remained dominant. Wetzels et al. (2018) found that *Megasphaera elsdenii* was dominant in abundance in RUSITEC samples as well as other *Lactobacillus* genera as seen in this experiment. Therefore, the effects observed in this study may not be replicated *in vivo* due to the differences in dominant bacterial populations.

Among the abundant genera (>0.1%) in the SAM samples, *Fibrobacter* was reduced in the 3.0 g HS/d treatment, but only on day 15. *Fibrobacter* spp. are fibrolytic and are commonly associated with the rumen where they produce succinate, formate, and acetate from plant-based cellulose (Weimer, 1993). Interestingly, Fernandes et al. (2015) noted a reduction in cellulose hydrolysis by *Fibrobacter succinogenes in vitro* when exposed to 0.05–5.0 g/L of HS. In the LAM samples, *Christensenellaceae* R-7 was reduced in the 3.0 g HS/d treatment. The *Christensenellaceae* family has been reported to be associated with low pH in the rumen of dairy cattle (De Nardi et al., 2016) and may play a role in the degradation of forage (Shen et al., 2017). *Escherichia* and *Methanobacterium* were the two genera that were most positively associated with the 3.0 g HS/d treatment. Although *Methanobacterium* was enriched in the HS treatment, the overall relative abundance of the class *Methanobacteria* did not differ and the other two methanogenic genera detected, *Methanobrevibacter* and *Methanosphaera*, were decreased by HS.

## Conclusion

The addition of HS to the diet had no effect on VFA production, DM disappearance, CH_4_ production, or the structure of the rumen microbiota. However, ammonia was decreased and the specific archaeal and bacterial genera were altered. Furthermore, HS at 3.0 g/d decreased the richness and diversity of the microbial community. In conclusion, the concentrations and type of bio-efficacy of the HS used did not appear to be a viable natural additive in ruminants to reduce CH_4_ production in the evaluated diet, however, there may be positive implications for HS in nitrogen metabolism.

## Author Contributions

AC, GB, TM, and ST: study design. ST, AR, and AC: conducting Rusitec study. ST, AR, DH, GB, and AC: lab analysis. ST and AR: DNA extraction. DH: bioinformatics. ST, AR, DH, TM, GB, and AC: wrote the manuscript. All authors read and approved the final manuscript.

## Conflict of Interest Statement

The authors declare that the research was conducted in the absence of any commercial or financial relationships that could be construed as a potential conflict of interest.
