# Supplementary material for: Humic Substances Alter Ammonia Production and the Microbial Populations Within a RUSITEC Fed a Mixed Hay – Concentrate Diet
Source: Front Microbiol. 2018 Jul 2;9:1410. doi: 10.3389/fmicb.2018.01410 (PMC6036602; doi:10.3389/fmicb.2018.01410)
Supplement: Supplementary file 1 [file Image_1.PDF]

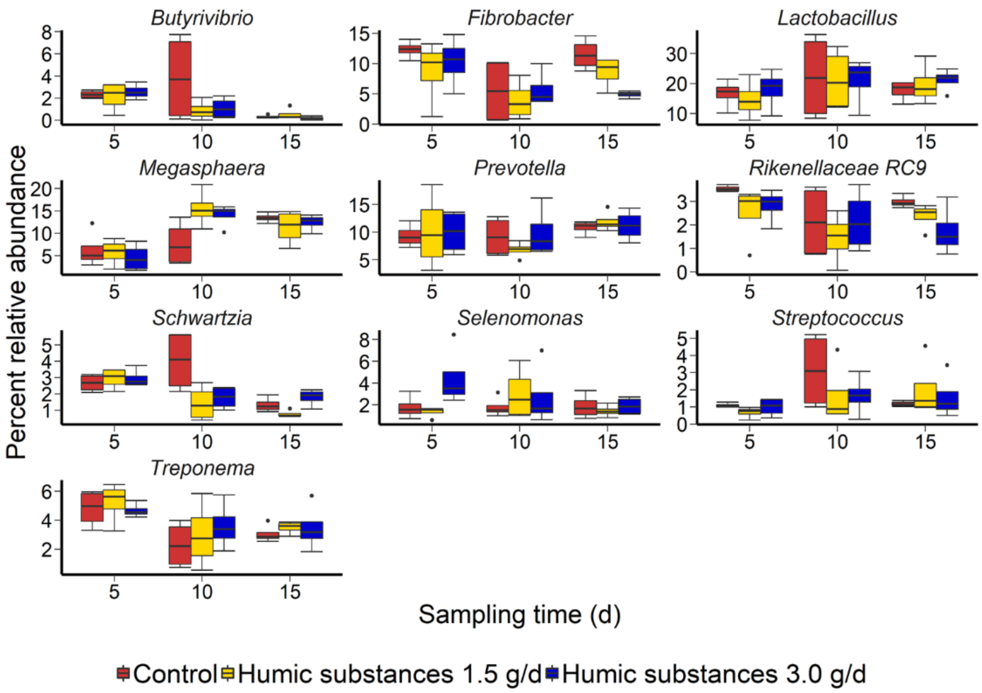

Supplementary Fig. S1. Relative abundance (%) of the ten most abundant genera in the solid-associated microbe (SAM) samples by treatment and sampling time. Points represent outlier samples.

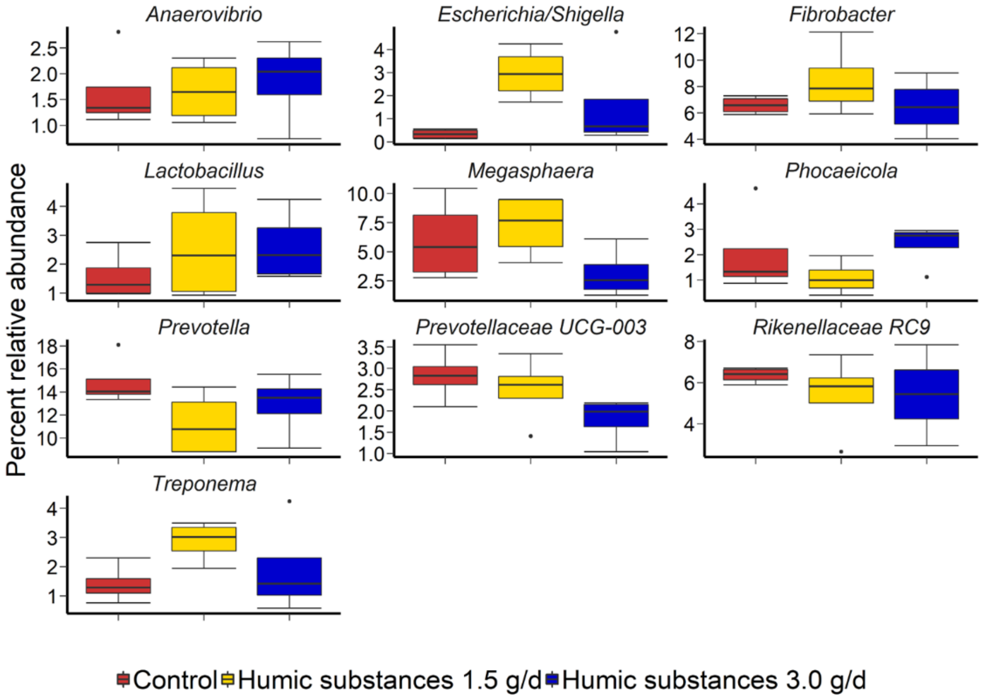

Supplementary Fig. S2. Relative abundance (%) of the ten most abundant genera in the liquid-associated microbe (LAM) samples by treatment (d 15). Points represent outlier samples.

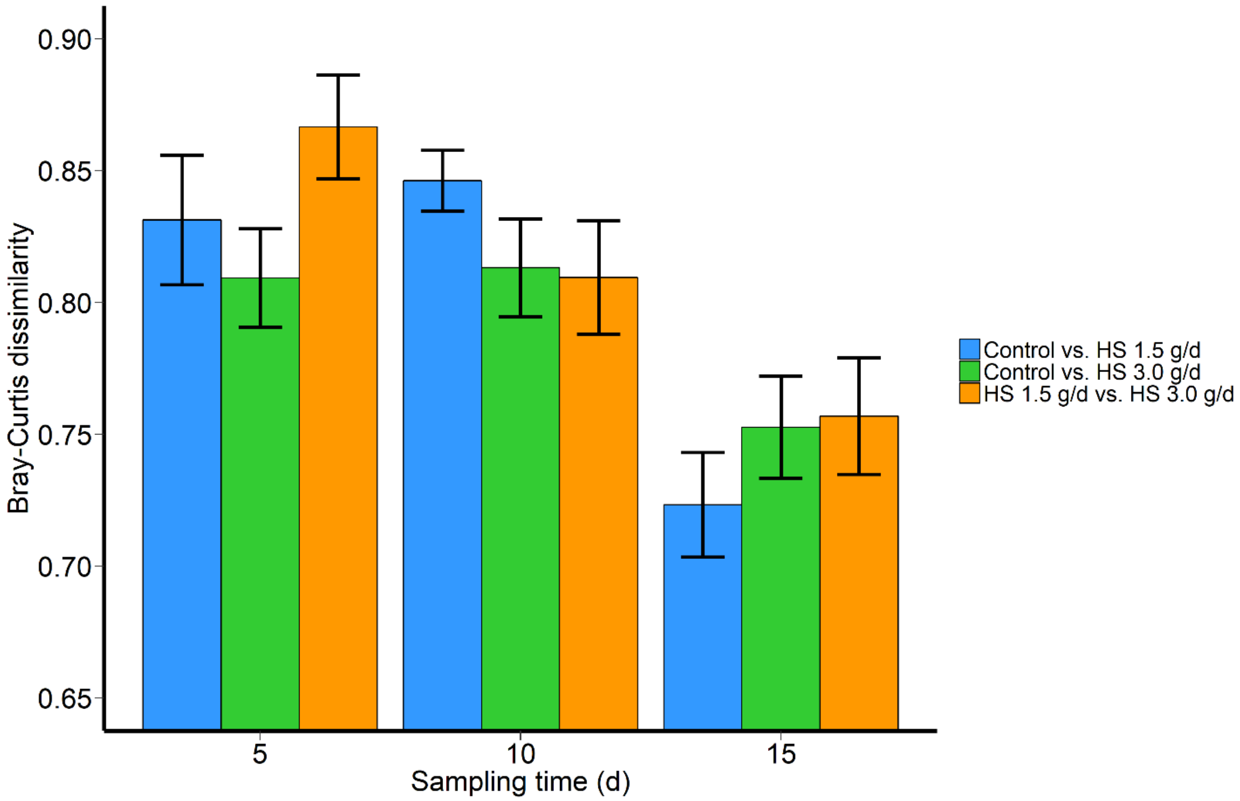

Supplementary Fig. S3. Bray-Curtis dissimilarities for pairwise treatment comparisons of solid-associated microbe (SAM) samples by sampling time. Error bars represent standard error of the mean (n = 16 comparisons). HS = humic substances.

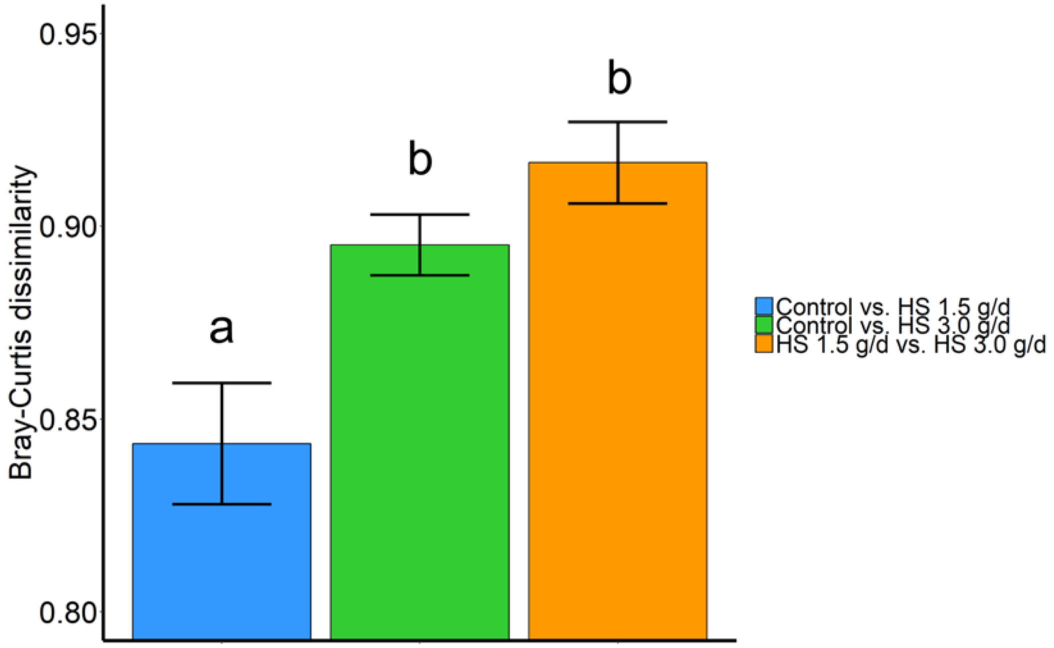

Supplementary Fig. S4. Bray-Curtis dissimilarities for pairwise treatment comparisons of liquid-associated microbe (LAM) samples taken at d 15. Error bars represent standard error of the mean (n = 16 comparisons). Different lowercase letters indicate significantly different means ($P < 0.05$). HS = humic substances.
